# Supplementary material for: Transient formation of supramolecular complexes between hyaluronan and oligopeptides at submicromolar concentration
Source: Commun Chem. 2026 Jan 13;9:34. doi: 10.1038/s42004-025-01834-2 (PMC12823696; doi:10.1038/s42004-025-01834-2)
Supplement: Supplementary file 2 — Supplementary information [file 42004_2025_1834_MOESM2_ESM.pdf]

Supplementary Information:

Transient Formation of Supramolecular Complexes Between Hyaluronan and Oligopeptides at Submicromolar Concentration

Miguel Riopedre-Fernandez,[†,#] Bingxin Chu,[‡,#] Anna Kuffel,[¶,§,#] Arianna Marchioro,[*,‡] Denys Biriukov,[*,‖,⊥,†] and Hector Martinez-Seara[*,†]

†*Institute of Organic Chemistry and Biochemistry of the Czech Academy of Sciences, Flemingovo nam. 542/2, CZ-160 00 Prague 6, Czech Republic*

‡*Laboratory for fundamental BioPhotonics (LBP), Institute of Bioengineering (IBI), School of Engineering (STI), Ecole polytechnique fédérale de Lausanne (EPFL), CH-1015 Lausanne, Switzerland*

¶*Faculty of Chemistry, Gdańsk University of Technology, Narutowicza 11/12, 80–233 Gdańsk, Poland*

§*BioTechMed Center, Gdańsk University of Technology, Narutowicza 11/12, 80–233 Gdańsk, Poland*

‖*Central European Institute of Technology, Masaryk University, Kamenice 5, 62500 Brno, Czech Republic*

⊥*National Centre for Biomolecular Research, Faculty of Science, Masaryk University, Kamenice 753/5, CZ-62500 Brno, Czech Republic*

#*These authors contributed equally to this work and are allowed to change the publication order to list them as first in their CVs*

E-mail: arianna.marchioro@epfl.ch; denysbiriukov@gmail.com; hseara@uochb.cas.cz

Molecular structures

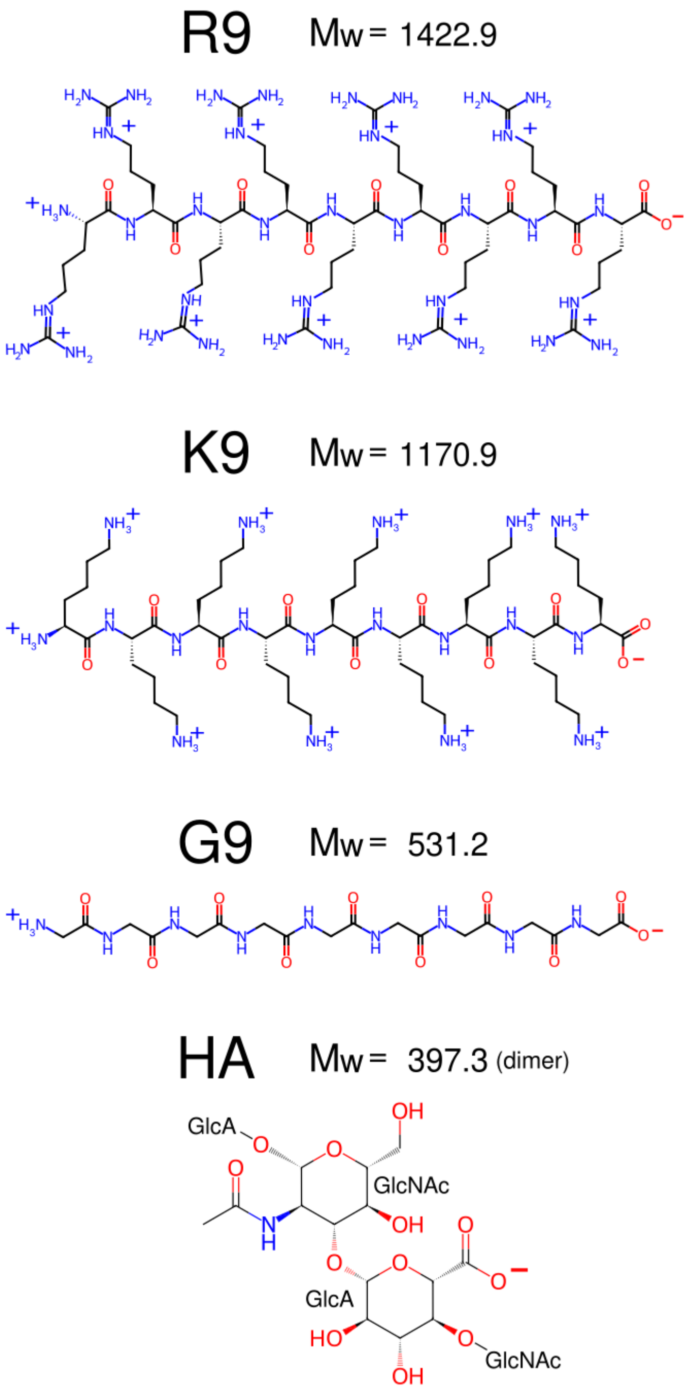

Supplementary Figure 1: Structures for R9, K9, G9, and HA with their expected protonation states at physiological conditions. The masses of the peptides correspond to the monoisotopic mass of the neutral species. The mass of the HA corresponds to that of an isolated disaccharide repeat unit.

NMR Meassurements

NOESY spectra on 8–15 KDa HA–nonapeptide mixtures

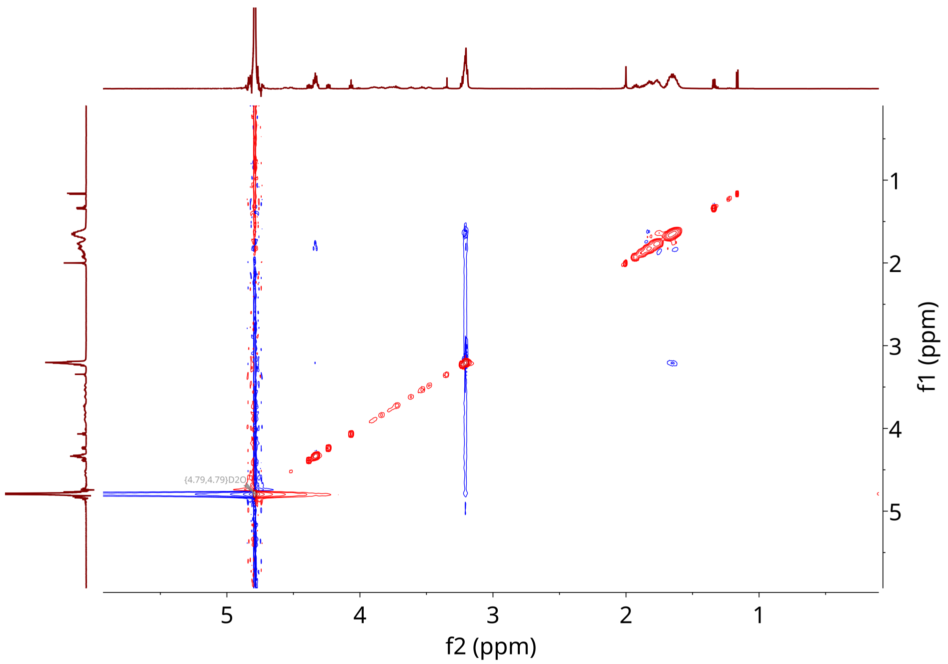

Supplementary Figure 2: The NOESY spectrum for the HA–R9 mixture with 8–15 KDa HA.

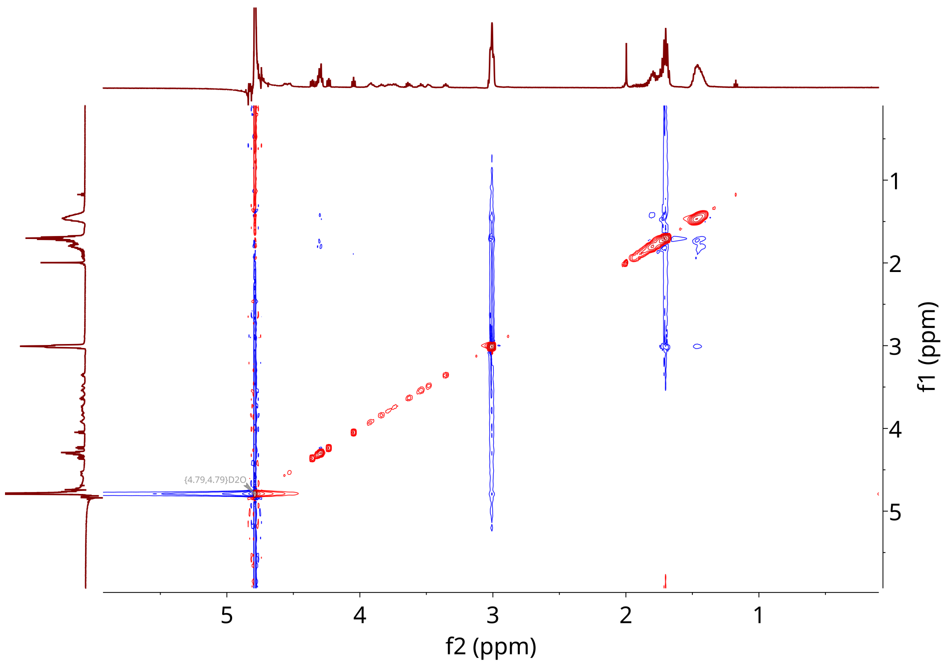

Supplementary Figure 3: The NOESY spectrum for HA–K9 mixture with 8–15 KDa HA.

NMR spectra on supernatants from 1,375 KDa HA–nonapeptide mixtures

Supplementary Note 1

We recorded the ^{1}H spectra of the solution supernatants prepared at different HA disaccharide/amino acid ratios, namely 0.87:1, 1.74:1, and 3.48:1, Figure 4. Already at a 1.74:1 ratio, the supernatants for R9 and K9 solutions do not contain HA signals, meaning that all the HA has precipitated out. Yet, R9 and K9 signals are always present, and their concentration increases non-monotonically as more peptide is added, indicative of a significant binding constant (see Figure 5). While a straight line would indicate that all the peptide was free in solution, the observed deviations from linearity mean that a fraction of the peptide material is incorporated into the precipitate. These behaviors indicate an equilibrium between the peptide and the precipitate, with the latter containing most or all the HA in the mixture. The higher abundance of R9 in the supernatant compared to K9 (Figure 5) may indicate a stronger interaction and faster saturation of binding sites by R9.

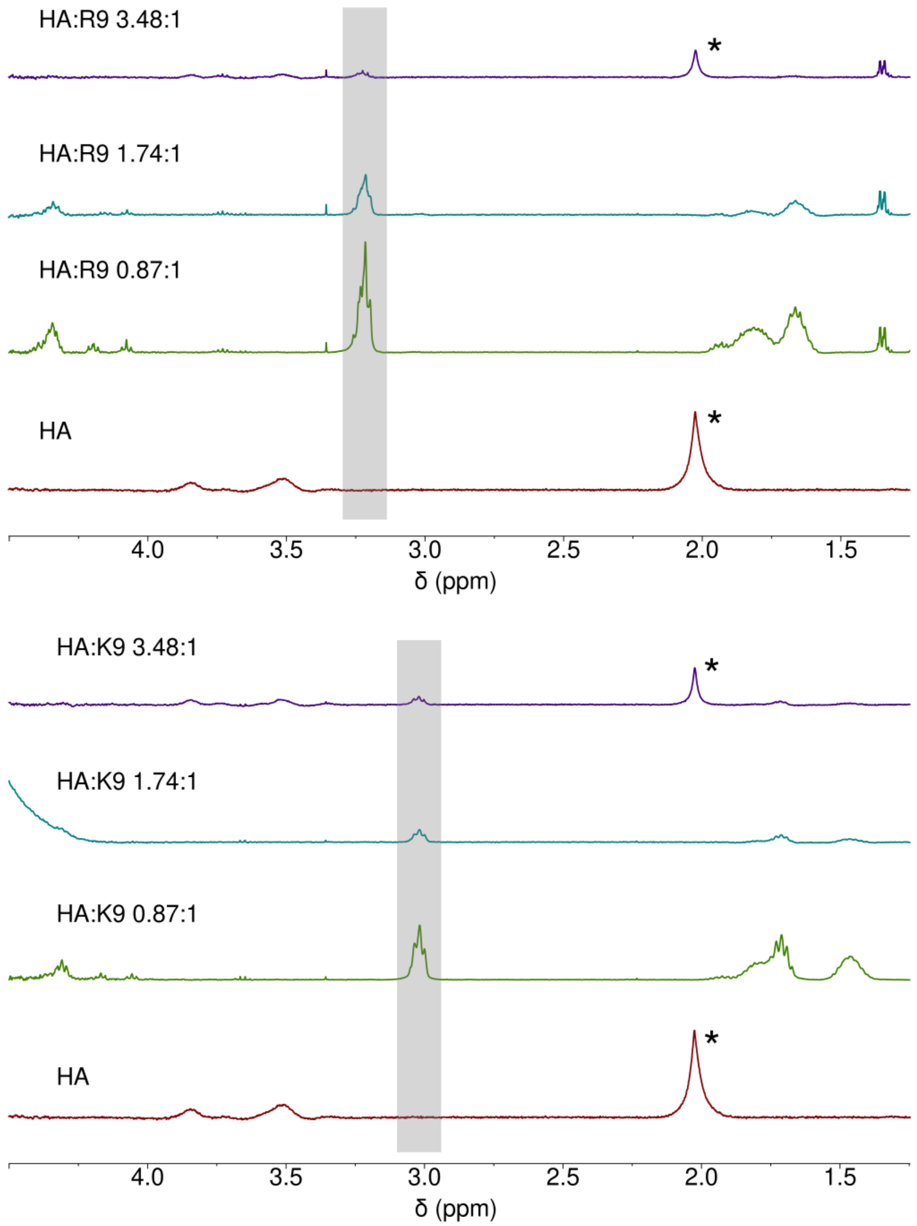

Supplementary Figure 4: Stacked ^{1}H NMR spectra of the supernatants at different HA dimer-to-amino acid ratios. The bottom trace corresponds to pure HA, while the others represent mixtures with R9 or K9 at varying concentrations. The gray-shaded region highlights the approximate area used for the integration of the peptide signal, selected for its clear and isolated peptide signal. One easily distinguishable signal, assigned to the acetamide group, is marked with an asterisk and was used to monitor HA's disappearance and subsequent reappearance in the solution. The spectra are produced in Mnova.[S1]

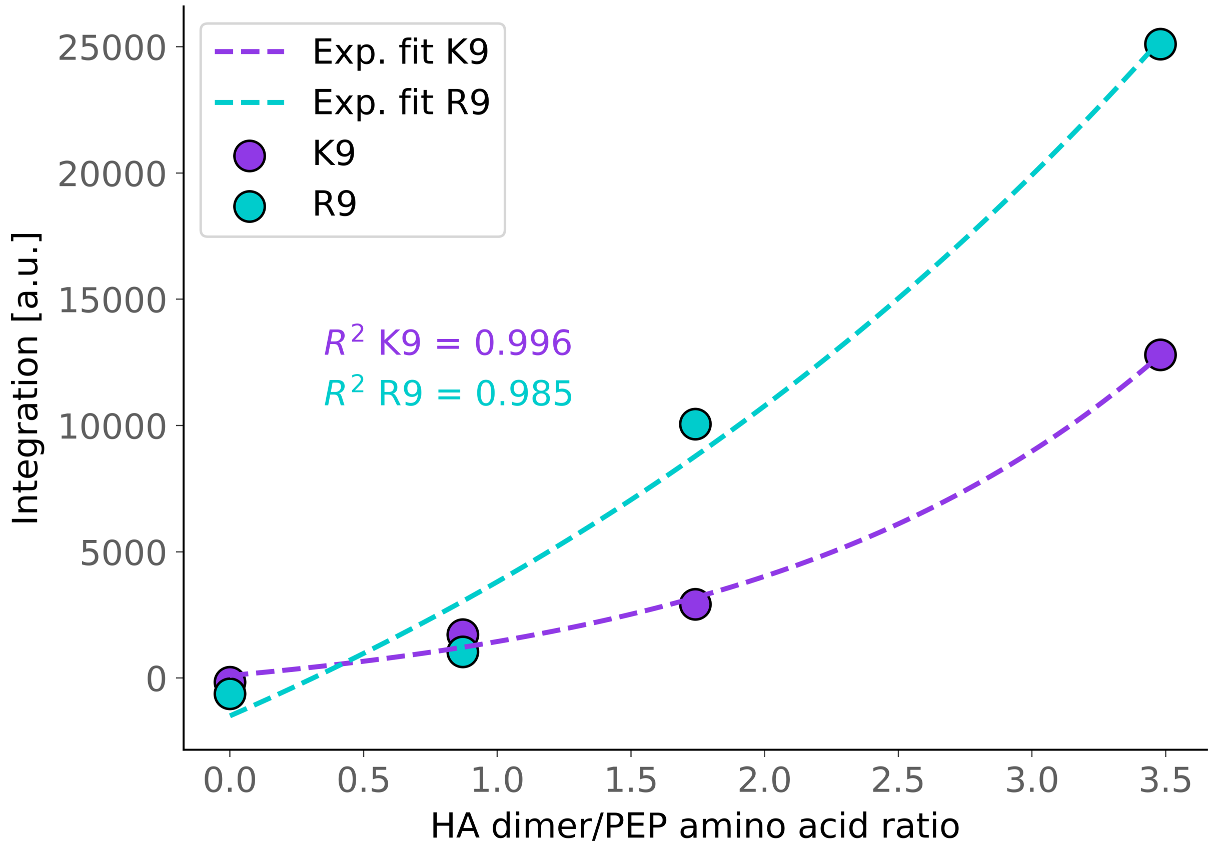

Supplementary Figure 5: Integrals of the observable peptide signals in the supernatant (integration between 3.3–3.1 ppm for R9 and 3.1–2.9 ppm for K9, respectively) as a function of the HA dimer-to-amino acid ratio. The integrals were calculated in Mnova,[S1] with baselines being adjusted using Whittaker smoothing, which rendered clean, flat baselines in the integration region. The resulting data points where then fitted to an exponential function of the form $A \times e^{b \times x} + c$.

Supplementary solution images

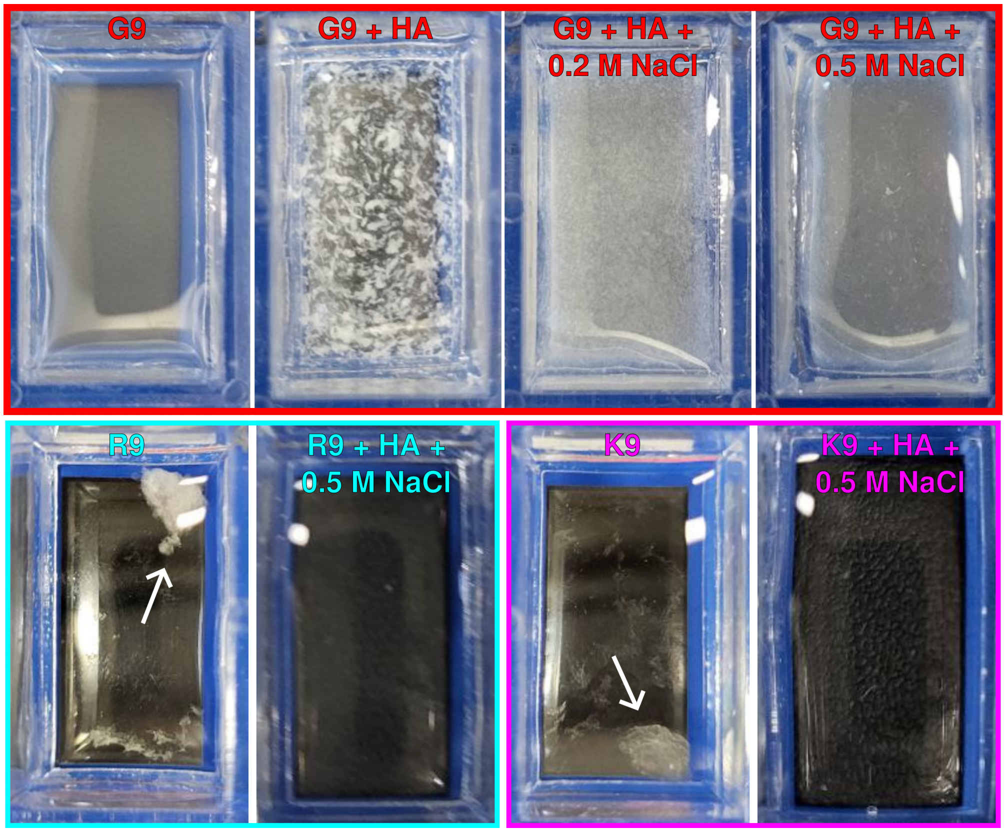

Supplementary Figure 6: (Top) Unmagnified views of a G9 solution, a G9 solution containing 1,375 kDa HA, and the same solution with two NaCl concentrations (0.2 M and 0.5 M). (Bottom) Unmagnified views of a R9 + HA solution, R9 + HA solution + 0.5 M NaCl, K9 + HA solution and K9 + HA solution + 0.5 M NaCl. The R9 + HA and K9 + HA can also be seen in Figure 1. Notice that the presence of 0.5 M NaCl stops the aggregation in all cases. The turbidity in the G9 + HA + NaCl is similar to that of the pure G9 and is caused by its low solubility. The red, turquoise and purple squares group the G9, R9 and K9 samples, respectively.

Supplementary AR-SHS data

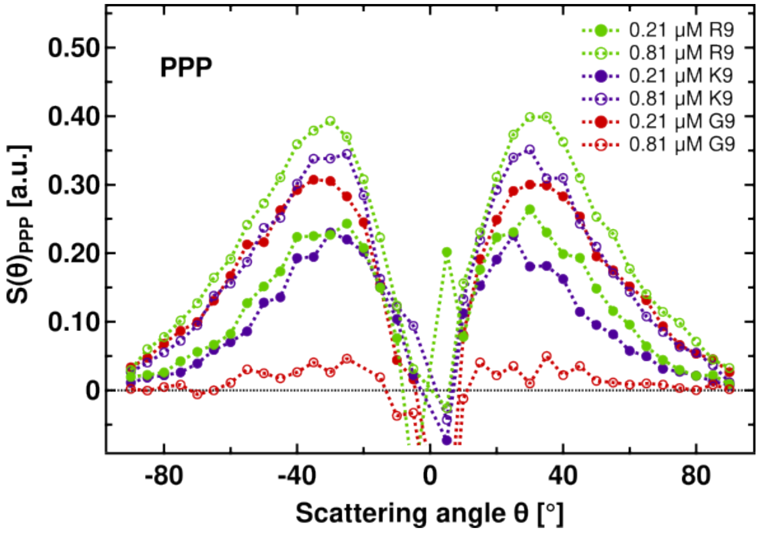

Supplementary Figure 7: Normalized AR-SHS patterns in the PPP polarization combination of pure peptide solutions. The normalization is done following Eq. 1, using water as a reference. The concentration of peptides in the reference solution is equal to the one in the mixture.

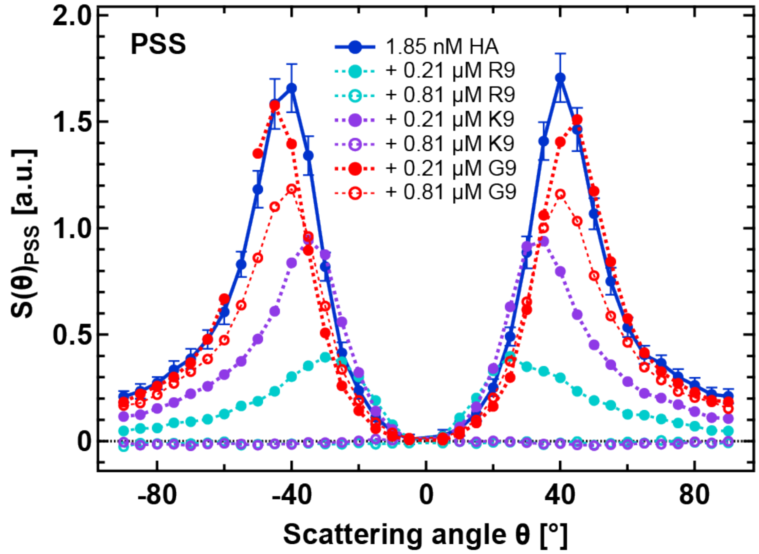

Supplementary Figure 8: Normalized AR-SHS patterns in the PSS polarization combination of a HA solution (1,375 kDa) and HA–peptide mixtures. The HA solution had a concentration of 1.85 nM, and each peptide—either R9, K9, or G9—is added at two different concentrations: 0.21 μM and 0.81 μM. The normalization is done following Eq. 1, using water as a reference for the HA solution and aqueous solutions of peptides as a reference for the mixtures. The concentration of peptides in the reference solution is equal to the one in the mixture. The error bars are shown for pure HA. These error bars represent the error propagation calculated for the normalized patterns, based on the standard deviation of 20 measurements.

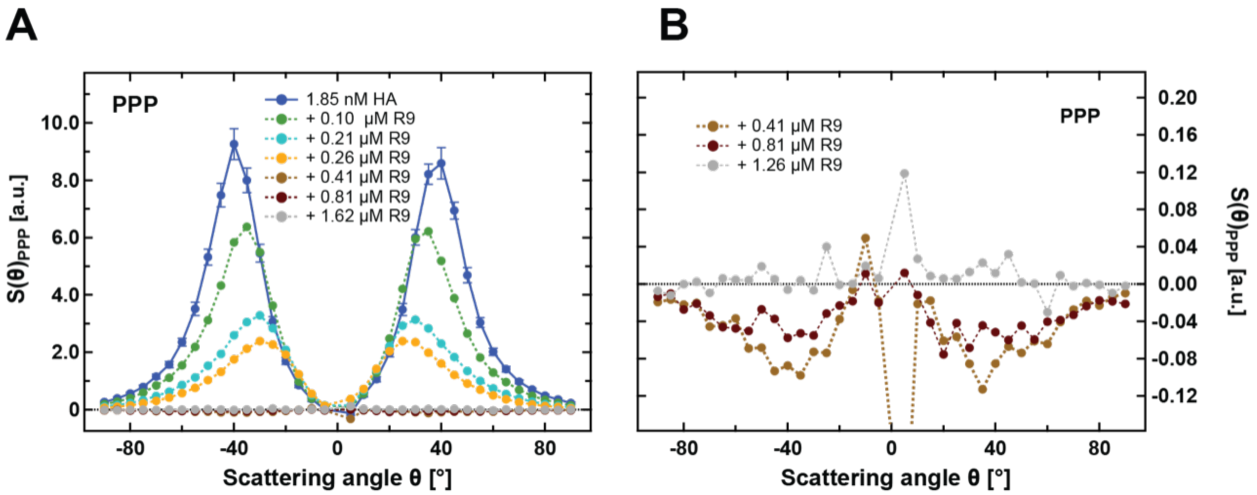

Supplementary Figure 9: A) An extended series of normalized AR-SHS patterns in the PPP polarization combination of a HA solution (1,375 kDa) and HA–R9 mixtures. The HA solution has a concentration of 1.85 nM, and the mixtures include R9 at varying concentrations. The normalization is done following Eq. 1, using water as a reference for the HA solution and aqueous solutions of peptides as a reference for the mixtures. The concentration of peptides in the reference solution is equal to that in the mixture. The error bars are shown for pure HA. These error bars represent the error propagation calculated for the normalized patterns, based on the standard deviation of 20 measurements. The effect of adding R9 to HA solutions on the pattern shape and intensity can already be seen at very low concentrations (0.1 μM). The effect is stronger with increasing R9 concentration. B) A zoomed-in view of the negative portion of the measured AR-SHS patterns at the highest R9 concentrations. A negative AR-SHS signal suggests that at the indicated concentrations, the mixtures induce less water orientation than the corresponding reference, here pure peptide solutions. Further studies would be necessary to fully understand the origin and the extent of this effect.

Conductivity and pH of AR-SHS solutions

Supplementary Note 2

The conductivity and pH were each measured once per sample and are reported in Table 1. Because we do not know the exact nature of the possible impurities, the measured conductivity cannot be converted to a known ionic concentration. However, one may estimate an upper limit for the possible ionic concentration by converting the maximum measured conductivity (5 µS/cm) using the molar ionic conductivity of an electrolyte with a typically low molar ionic conductivity, *e.g.*, KCl. This rough estimation indicates an upper limit of 20 µM of monovalent ions for the HA–peptide mixtures. Despite corresponding to a low amount of charged impurities, our optical experiment is sensitive to extremely small amounts of charged impurities that could participate in the decrease in the AR-SHS signal intensity.

Supplementary Table 1: Conductivity κ and pH of studied HA and HA–peptide solutions for AR-SHS experiments.

Sample	κ [µS/cm]	pH
HA 1.85 nM	1.7	6.3
HA 1.85 nM + R9 0.21 µM	1.8	6.1
HA 1.85 nM + R9 0.81 µM	4.2	6.6
HA 1.85 nM + K9 0.21 µM	3.2	5.9
HA 1.85 nM + K9 0.81 µM	2.7	5.9
HA 1.85 nM + G9 0.21 µM	1.4	6.7
HA 1.85 nM + G9 0.81 µM	1.8	6.2

Supplementary Molecular Dynamics Data

Solvent-accessible surface area

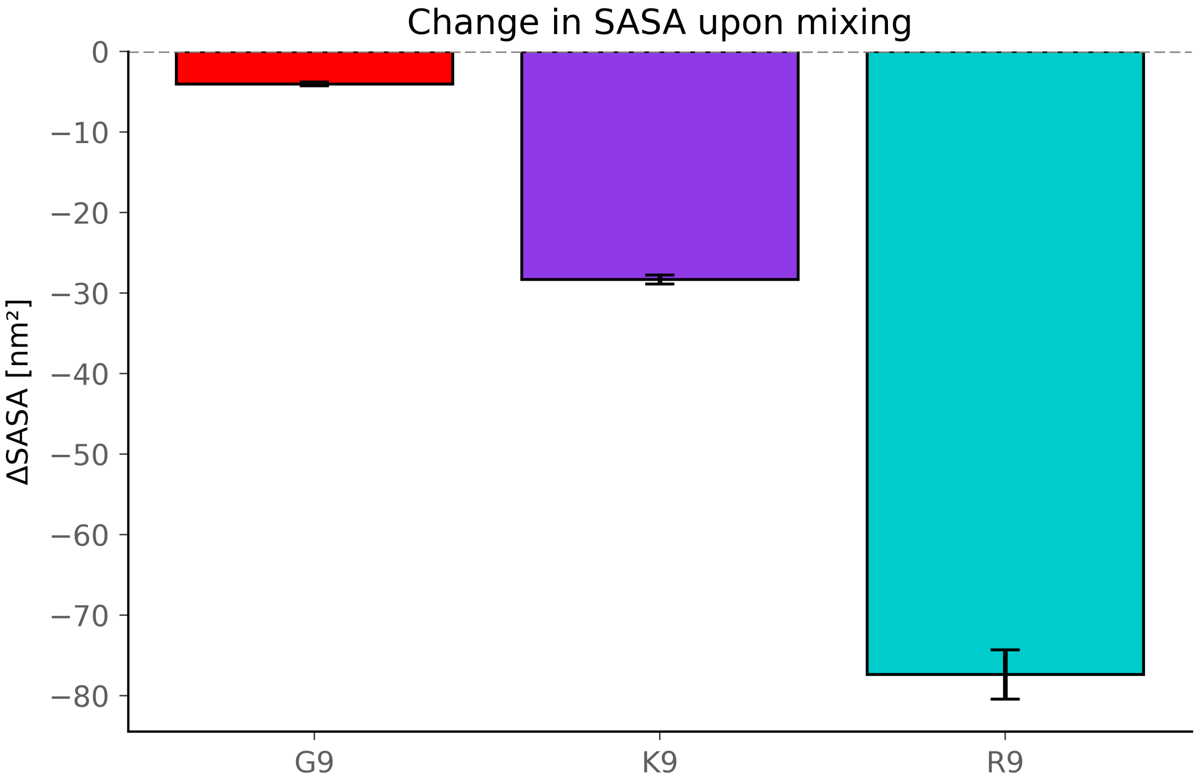

Supplementary Figure 10: Change in the solvent-accessible surface area (SASA) of the solutes (HA and/or peptides) upon mixing, compared to the combined SASA from separate simulations of each component. The difference is calculated as $\Delta SASA = SASA_{mix} - (SASA_{HA} + SASA_{pep})$. The SASA values were calculated using `gmx sasa` tool in GROMACS software, version 2023.1,[S2] with default parameters. The plot displays the average values across all replicas and the corresponding standard error of the mean.

Aggregate characterization

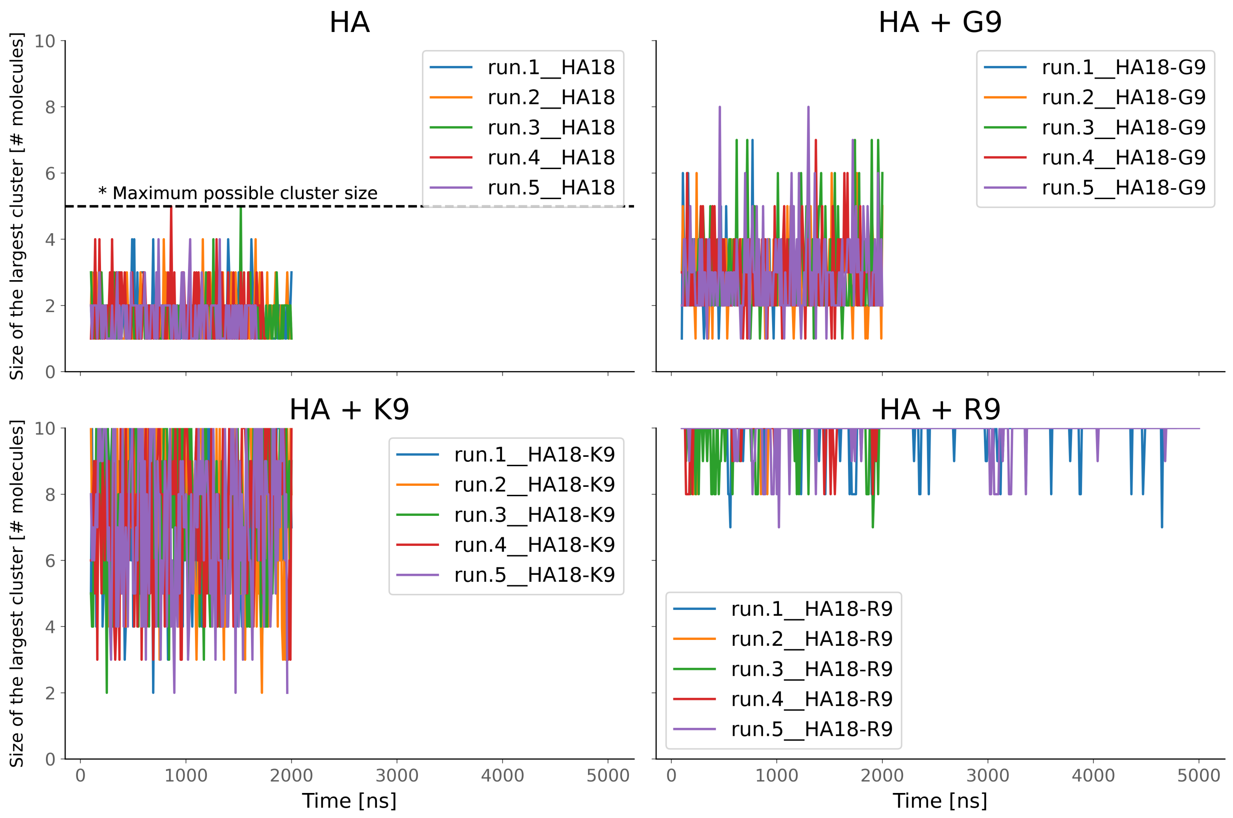

Supplementary Figure 11: Time evolution of the size of the largest cluster. All replicas for each system type are shown. The size of the largest cluster is defined as the number of solute molecules (HA and/or peptides) in continuous contact, with a cutoff of 3.5 Å.

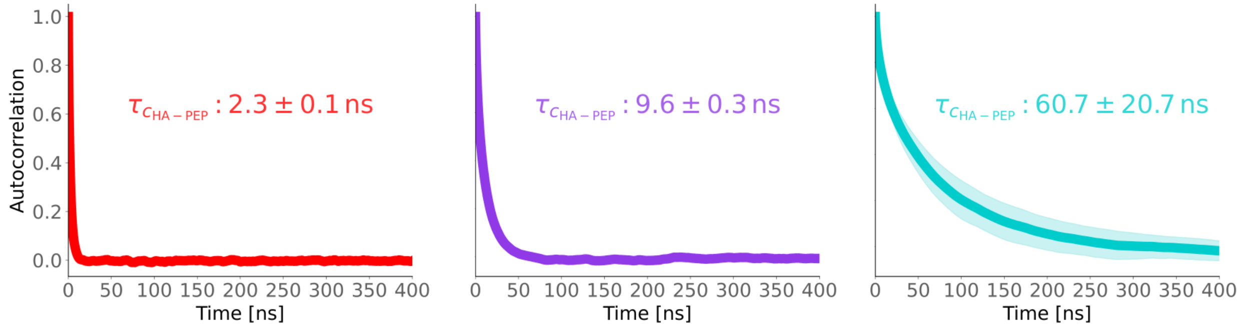

Supplementary Figure 12: HA–peptide distance autocorrelation function. The minimum distance between every residue pair was included in the time series. The autocorrelation characteristic times are calculated by fitting the data to an exponential function of the form e^{-t/τ_c}, where τ is the lag time. The standard deviation—calculated for all simulation replicas and shown as a shaded area—is notable only for HA–R9 systems.

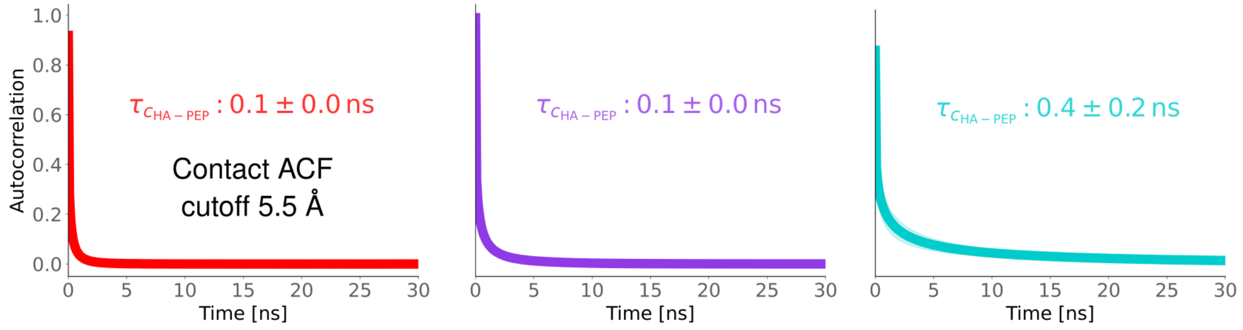

Supplementary Figure 13: HA–peptide contact autocorrelation function. The minimum distance between every residue pair was included in the time series. The HA–peptide residues center of mass distances are converted to contacts with a cutoff of 5.5 Å. The autocorrelation characteristic times are calculated by fitting the data to an exponential function of the form e^{-t/τ_c}, where τ is the lag time. The standard deviation is calculated for all simulation replicas and shown as a shaded area.

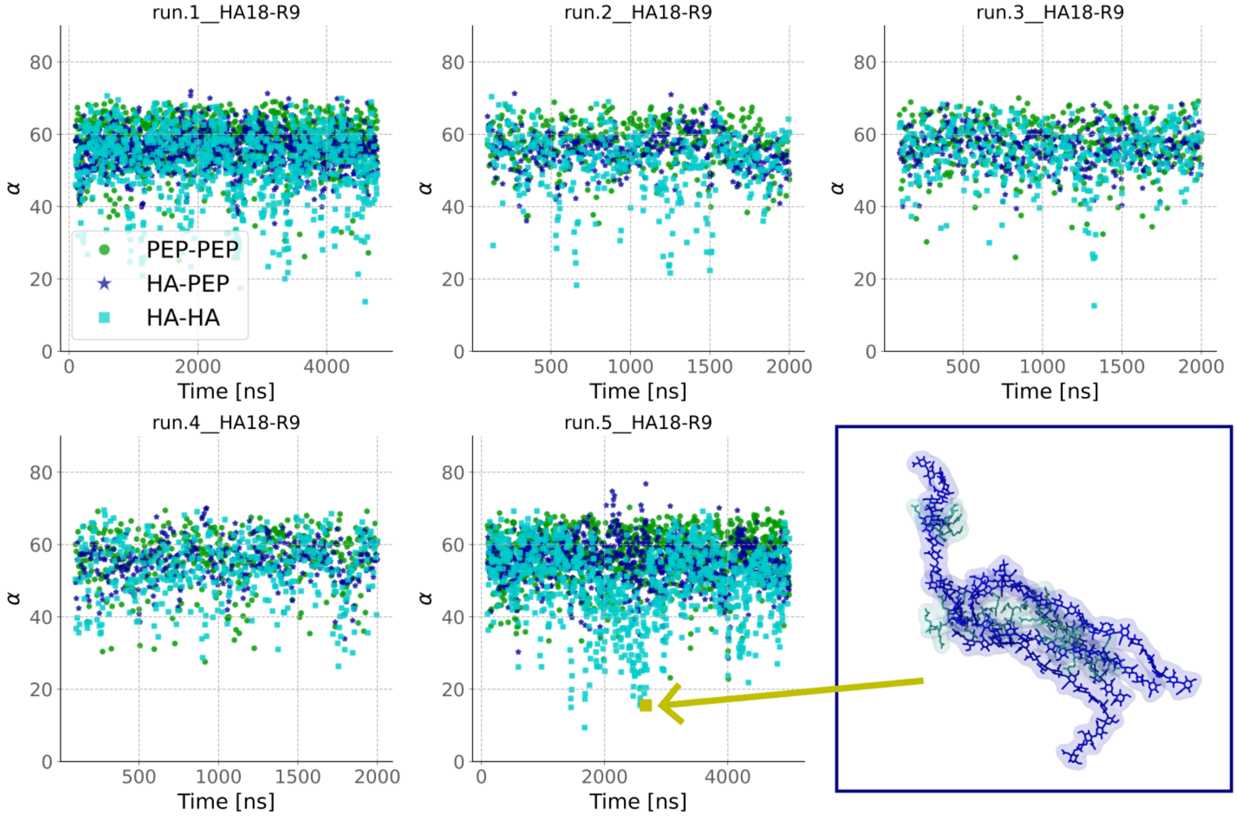

Supplementary Figure 14: Time evolution of the average angles between the molecules in the HA–R9 systems. All five replicas are shown independently, and the HA–HA, HA–PEP, and PEP–PEP are shown. The angles shown are the average angle between all pairs of molecules of a given type. The molecular axis used for the angle analysis was determined using singular value decomposition. A snapshot from a frame with low HA–HA angle values is included to illustrate the bundled structures associated with these low-angle configurations. These bundles form and dissociate spontaneously throughout the simulations and are not observed in the K9 or G9 systems. The HA molecules are shown in blue, while the R9 is shown in cyan. A yellow dot and arrow highlight the specific frame from which the snapshot was taken.

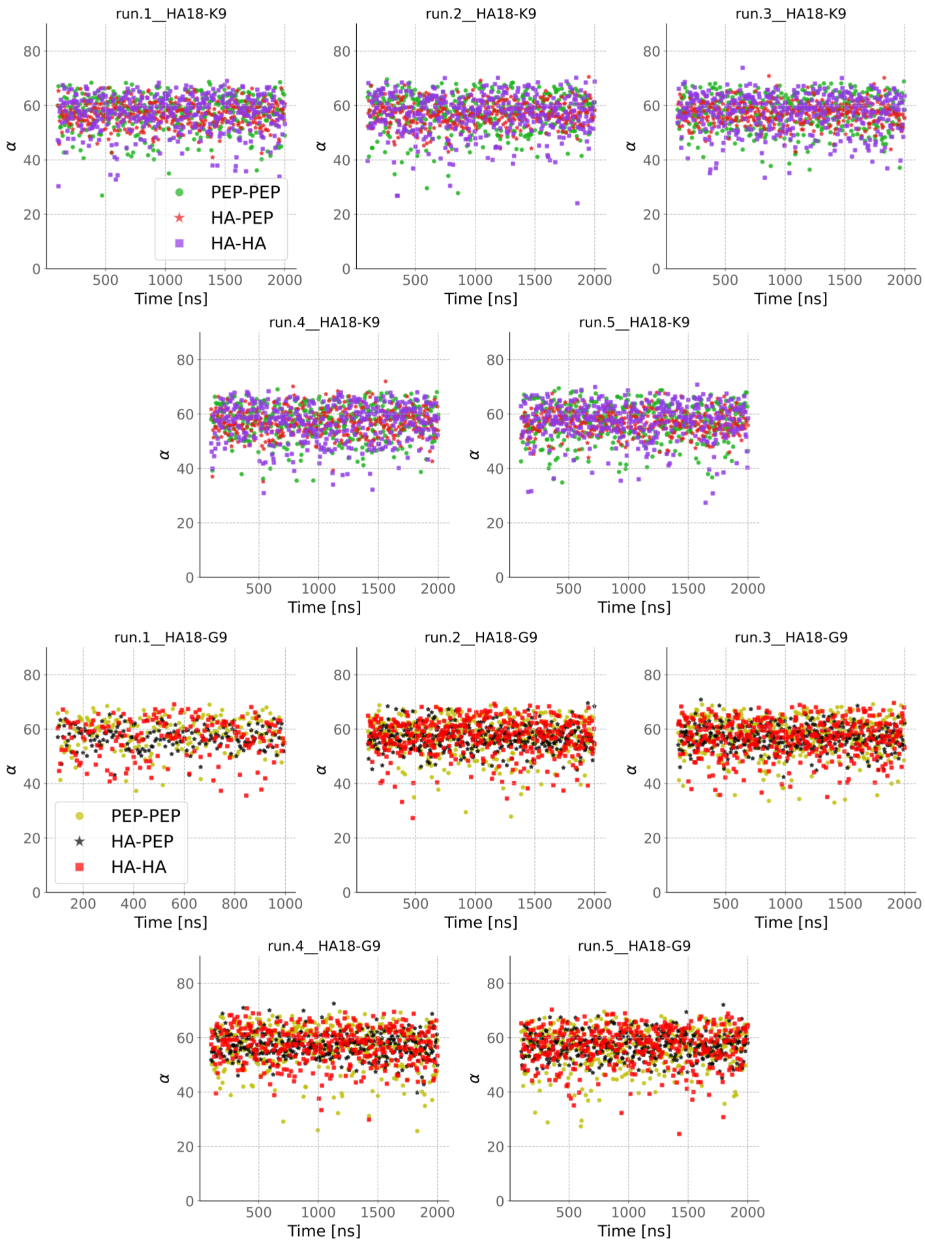

Supplementary Figure 15: Time evolution of the average angles between the molecules in the HA–K9 (purple) and HA–G9 (red) systems. All five replicas are shown independently, and the HA–HA, HA–PEP and PEP–PEP are shown. The angles shown are the average angle between all pairs of molecules of a given type. The molecular axis used for the angle analysis was determined using singular value decomposition.

Orientation of water molecules with respect to the solutes

Supplementary Note 3

Water orientation is calculated as the angle between the water bisector vector and the vector pointing from the water oxygen to the nearest solute atom. Since water is in the liquid state, it is highly mobile, and when averaging over many configurations, an angle of 90° indicates random orientation. Angles less than 90° indicate that, on average, the water hydrogen atoms are oriented toward the solute. In comparison, angles greater than 90° suggest that the water oxygen tends to be closer to the solute.

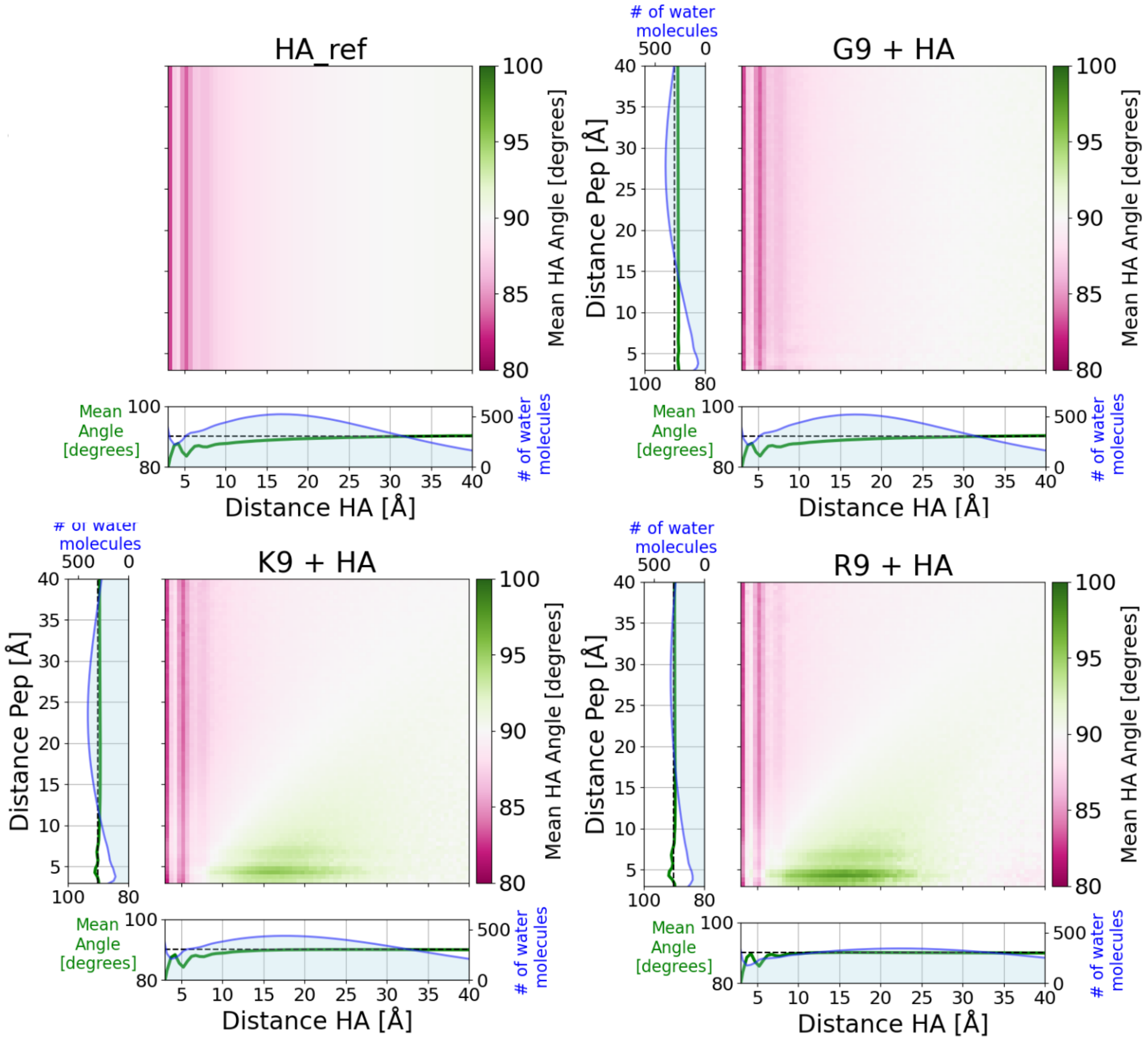

Supplementary Figure 16: Absolute average water orientations with respect to HA as a function of the distance to both HA and peptides. The orientation of water molecules was calculated as the angle between the water bisector and the vector connecting the water oxygen to the nearest atom of an HA molecule. These data are used as a reference for the normalization in Figure 4B in the main text. In the pure HA system (top left), there are no peptides, so the vertical axis is constant. Marginal plots show the average angle in each bin and the average number of water molecules per frame in that bin. These data contain the average of all applicable replicates.

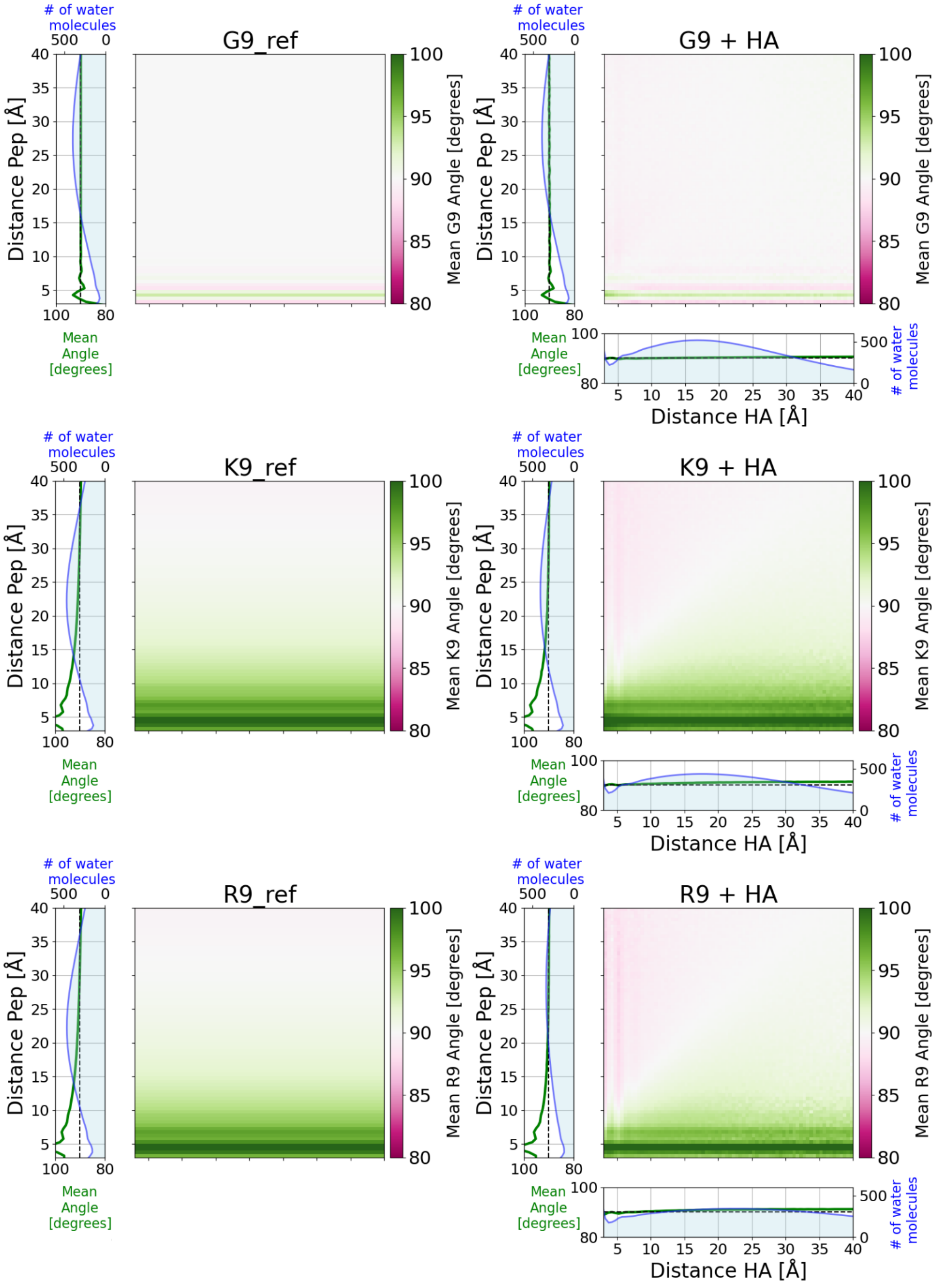

Supplementary Figure 17: Absolute average water orientations with respect to peptides as a function of the distance to both HA and peptides. The orientation of water molecules was calculated as the angle between the water bisector and the vector connecting the water oxygen to the nearest atom of a peptide molecule. These data are used as a reference for the normalization in Figure 4C in the main text. In the pure peptide systems (left column), there is no HA, so the horizontal axis is constant. Marginal plots show the average angle in each bin and the average number of water molecules per frame in that bin. These data contain the average of all applicable replicates.

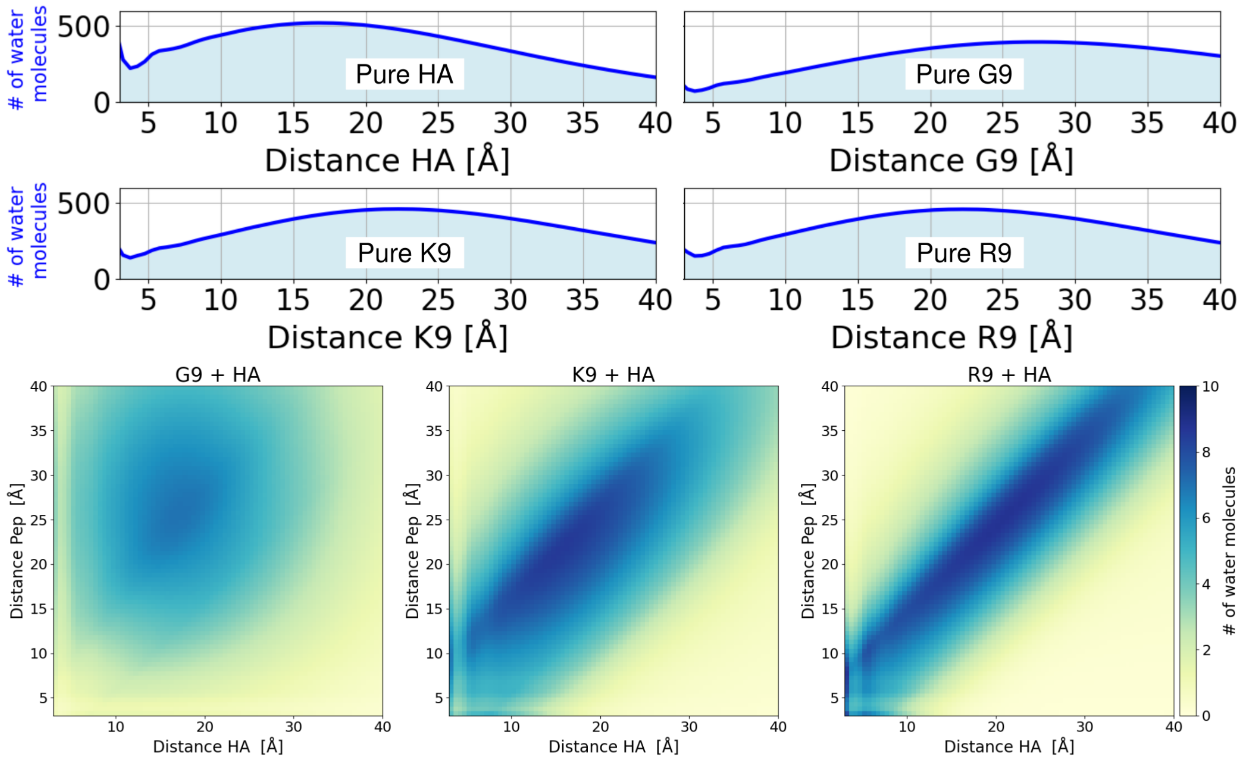

Supplementary Figure 18: Number of water molecules as a function of distance to HA or the corresponding peptide. The two upper rows show the distribution of water molecules in pure solutions. The 2D plots show the same for the HA–peptide mixtures. These data contain the average of all applicable replicates.

Supplementary peptide characterization data

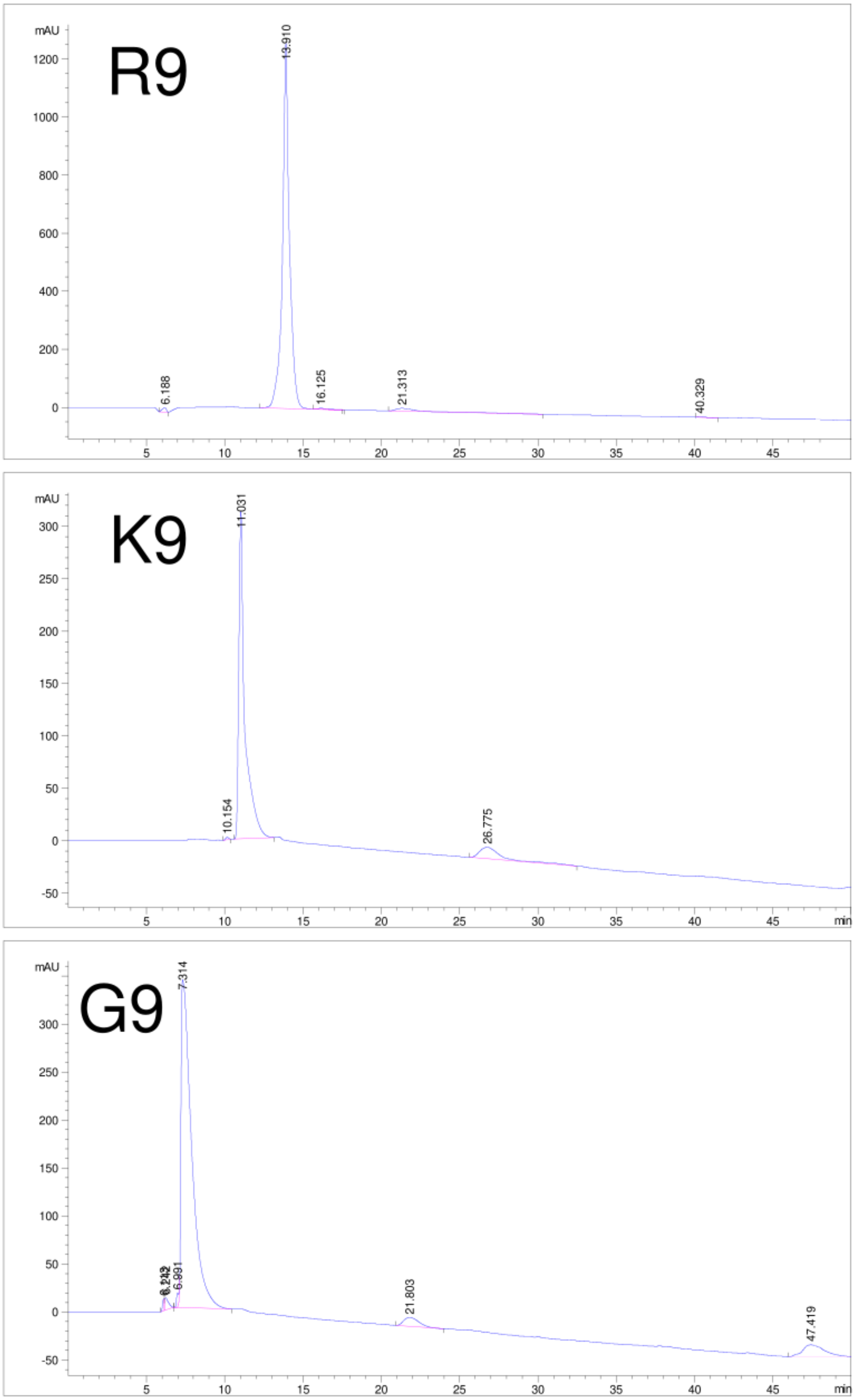

Supplementary Figure 19: HPLC traces for R9, K9, and G9 peptides.

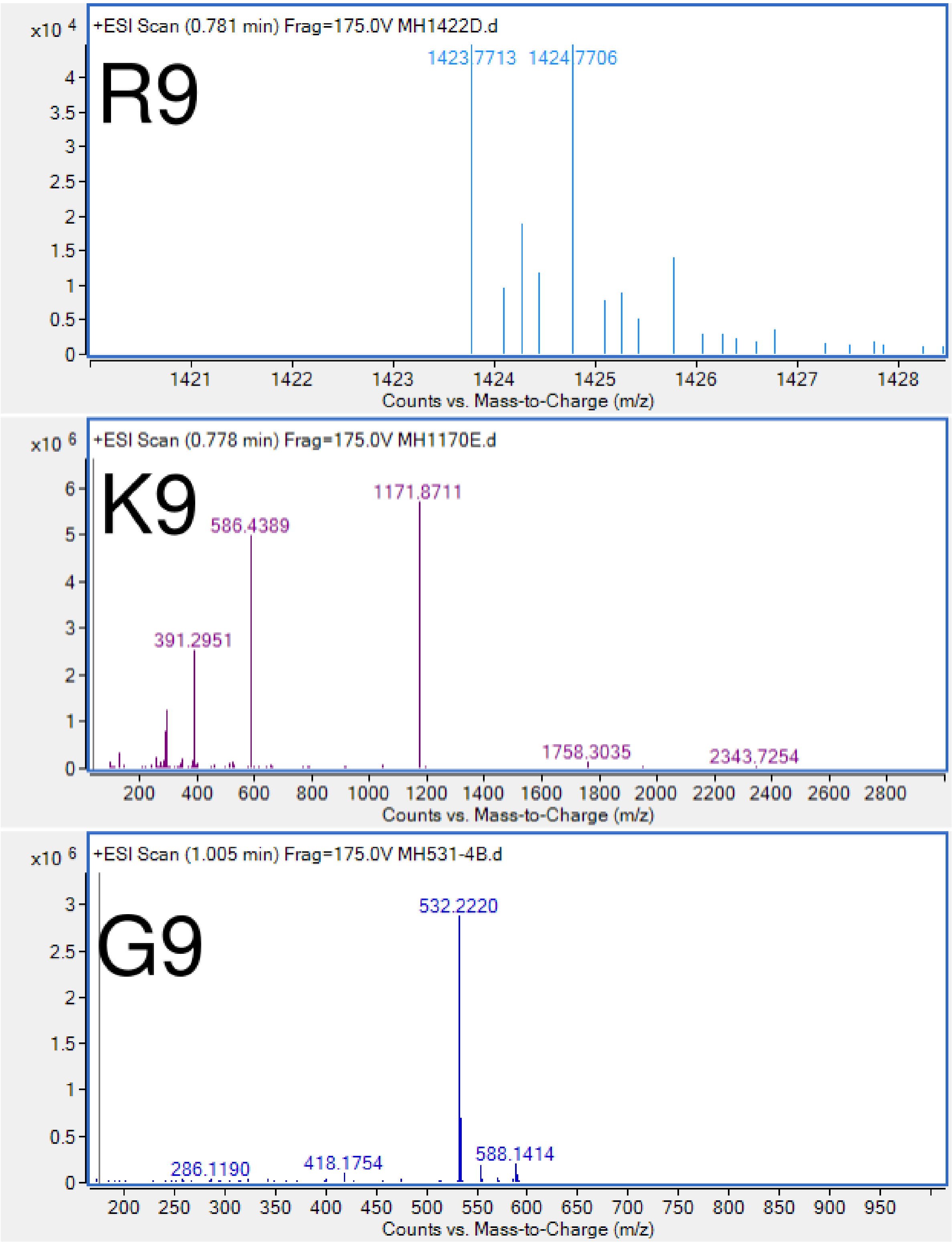

Supplementary Figure 20: Mass spectrometry (MS) traces for R9, K9, and G9 peptides. All peptides exhibit high purity, with signals corresponding exclusively to their expected molecular ions. The corresponding main peaks are: R9 [M+H]+ = 1423.8, K9 [M+H]+ = 1171.9, G9 [M+H]+ = 532.2. Minor peaks correspond to isotopic variants or other fractionation products.

Supplementary References

(S1) Willcott, M. R. MestRe Nova. *J. Am. Chem. Soc.* **2009**, *131*, 13180–13180.

(S2) Abraham, M. J.; Murtola, T.; Schulz, R.; Páll, S.; Smith, J. C.; Hess, B.; Lindah, E. Gromacs: High performance molecular simulations through multi-level parallelism from laptops to supercomputers. *SoftwareX* **2015**, *1-2*, 19–25.
