# Supplementary material for: Transient formation of supramolecular complexes between hyaluronan and oligopeptides at submicromolar concentration
Source: Commun Chem. 2026 Jan 13;9:34. doi: 10.1038/s42004-025-01834-2 (PMC12823696; doi:10.1038/s42004-025-01834-2)
Supplement: Supplementary file 3 — Reporting summary [file 42004_2025_1834_MOESM3_ESM.pdf]

nature portfolio

Corresponding author(s): Arianna Marchioro, Denys Biriukov, Hector Martinez-Seara

Last updated by author(s): Nov 14, 2025

Reporting Summary

Nature Portfolio wishes to improve the reproducibility of the work that we publish. This form provides structure for consistency and transparency in reporting. For further information on Nature Portfolio policies, see our Editorial Policies and the Editorial Policy Checklist.

Statistics

For all statistical analyses, confirm that the following items are present in the figure legend, table legend, main text, or Methods section.

n/a	Confirmed	
☐	☒	The exact sample size (*n*) for each experimental group/condition, given as a discrete number and unit of measurement
☐	☒	A statement on whether measurements were taken from distinct samples or whether the same sample was measured repeatedly
☒	☐	The statistical test(s) used AND whether they are one- or two-sided<br>*Only common tests should be described solely by name; describe more complex techniques in the Methods section.*
☒	☐	A description of all covariates tested
☒	☐	A description of any assumptions or corrections, such as tests of normality and adjustment for multiple comparisons
☐	☒	A full description of the statistical parameters including central tendency (e.g. means) or other basic estimates (e.g. regression coefficient) AND variation (e.g. standard deviation) or associated estimates of uncertainty (e.g. confidence intervals)
☒	☐	For null hypothesis testing, the test statistic (e.g. *F*, *t*, *r*) with confidence intervals, effect sizes, degrees of freedom and *P* value noted<br>*Give P values as exact values whenever suitable.*
☒	☐	For Bayesian analysis, information on the choice of priors and Markov chain Monte Carlo settings
☒	☐	For hierarchical and complex designs, identification of the appropriate level for tests and full reporting of outcomes
☒	☐	Estimates of effect sizes (e.g. Cohen's *d*, Pearson's *r*), indicating how they were calculated

Our web collection on statistics for biologists contains articles on many of the points above.

Software and code

Policy information about availability of computer code

Data collection	GROMACS versions 2022.3 and 2023.1
Data analysis	GROMACS versions 2022.3 and 2023.1; Mnova version 15.0.1-1; python 3.13.7, numpy 2.3.4, matplotlib 0.2.1, Igor Pro version 7, Excel Microsoft 365, custom scripts

For manuscripts utilizing custom algorithms or software that are central to the research but not yet described in published literature, software must be made available to editors and reviewers. We strongly encourage code deposition in a community repository (e.g. GitHub). See the Nature Portfolio guidelines for submitting code & software for further information.

Data

Policy information about availability of data

All manuscripts must include a data availability statement. This statement should provide the following information, where applicable:

- Accession codes, unique identifiers, or web links for publicly available datasets
- A description of any restrictions on data availability
- For clinical datasets or third party data, please ensure that the statement adheres to our policy

The data and materials associated with this work are available on Zenodo under the DOI: 10.5281/zenodo.15115544 (a collection of microscope images and videos), 10.5281/zenodo.15115660 (all the simulations and files needed to replicate them), and 10.5281/zenodo.17122576 (NMR data and spectra).
All raw data used for the plots in the main text, including AR-SHS data, are available at Zenodo, DOI: 10.5281/zenodo.17611850.

Research involving human participants, their data, or biological material

Policy information about studies with human participants or human data. See also policy information about sex, gender (identity/presentation), and sexual orientation and race, ethnicity and racism.

Reporting on sex and gender	This study did not involve human participants, animals, or other biological subjects for which sex or gender is relevant. Therefore, reporting on sex and gender does not apply.
Reporting on race, ethnicity, or other socially relevant groupings	This study did not involve human participants or population-based data. Therefore, reporting on race, ethnicity, or other socially relevant groupings is not applicable.
Population characteristics	This study did not involve any human, animal, or other biological populations. Therefore, reporting of population characteristics is not applicable.
Recruitment	This study did not involve the recruitment of human participants, animals, or any biological subjects. Therefore, this section is not applicable.
Ethics oversight	This study did not involve human participants, animal subjects, or other ethically regulated materials. Therefore, ethics approval and oversight were not required, and this section is not applicable.

Note that full information on the approval of the study protocol must also be provided in the manuscript.

Field-specific reporting

Please select the one below that is the best fit for your research. If you are not sure, read the appropriate sections before making your selection.

☒ Life sciences ☐ Behavioural & social sciences ☐ Ecological, evolutionary & environmental sciences

For a reference copy of the document with all sections, see nature.com/documents/nr-reporting-summary-flat.pdf

Life sciences study design

All studies must disclose on these points even when the disclosure is negative.

Sample size	Experimental sample sizes were determined empirically based on reproducibility and signal-to-noise considerations, while the number of computational replicates was selected to ensure statistical convergence. No statistical methods were used to predetermine sample size.
Data exclusions	No data were excluded from the analyses.
Replication	Reproducibility was verified through independent repetitions and consistency checks. A single replicate was performed for NMR and microscopy imaging because these experiments provide high-fidelity outputs with minimal experimental variability. All other reported results were confirmed by multiple independent observations or calculations.
Randomization	Randomization was incorporated through independent initialization of simulations and random assignment of starting configurations to ensure unbiased sampling of system states. For experimental measurements, sample order and data acquisition were randomized where possible. All experiments were conducted under controlled conditions to ensure consistency across randomized runs.
Blinding	Blinding was not applicable, as all measurements and analyses were conducted using automated or objective procedures, leaving no opportunity for operator bias. Experimental and computational workflows were fully standardized.

Reporting for specific materials, systems and methods

We require information from authors about some types of materials, experimental systems and methods used in many studies. Here, indicate whether each material, system or method listed is relevant to your study. If you are not sure if a list item applies to your research, read the appropriate section before selecting a response.

Materials & experimental systems

n/a	Involved in the study
☒	☐ Antibodies
☒	☐ Eukaryotic cell lines
☒	☐ Palaeontology and archaeology
☒	☐ Animals and other organisms
☒	☐ Clinical data
☒	☐ Dual use research of concern
☒	☐ Plants

Methods

n/a	Involved in the study
☒	☐ ChIP-seq
☒	☐ Flow cytometry
☒	☐ MRI-based neuroimaging

Plants

Seed stocks	This study did not involve the use of seed stocks or any plant-derived biological materials.
Novel plant genotypes	This study did not involve plants or the generation or use of novel plant genotypes.
Authentication	This study did not involve plants or plant-derived materials, and no authentication procedures were required.
